# The value of enhanced ethics checks: Initial experiences with *PLOS ONE*’s updated Human Subjects Research policy

**DOI:** 10.1371/journal.pone.0288900

**Published:** 2023-08-09

**Authors:** Renee Hoch, Emily J. Chenette

**Affiliations:** 1 Managing Editor, PLOS Publication Ethics; 2 Editor in Chief, PLOS ONE; Public Library of Science, UNITED KINGDOM

Publisher’s note: This editorial was adapted from a 1 March 2023 post on the EveryONE PLOS blog: https://everyone.plos.org/2023/03/01/an-update-to-the-human-subjects-research-policy-on-plos-one/

*PLOS ONE* has always had high ethics standards for the research that we publish. In March 2023, *PLOS ONE* updated its Human Subjects Research Policy to require that authors provide ethics approval documentation at initial submission for studies reporting research involving human participants [1]. Here, we provide context for why we made this change and share our initial experiences with this updated policy.

Our publication criteria require that “research meets all applicable standards for the ethics of experimentation and research integrity” [2], which are described in more detail in our publication policies [3]. Moreover, compliance with PLOS policies is checked at multiple points by journal staff. This important work helps ensure that articles published by *PLOS ONE* meet all applicable national and international regulations, and adhere to high standards for research and publication ethics.

The competitive nature of academic publishing, tenure, and hiring decisions can incentivize researchers to take shortcuts, compromising on ethics to boost or accelerate their publication records [4]. In some cases, this might mean conducting research before all requisite approvals have been obtained. In others, it might mean purchasing authorship or article content, or compromising the integrity of the peer review process. These unethical practices can pollute the literature with problematic articles that are harmful to the broader community, including people endeavoring to replicate fraudulent research and those directly impacted by the published findings.

PLOS and other publishers have recently seen a rise in large-scale cases involving manipulation of the publication process [5–8], and we are working to increase the stringency of our processes to keep problematic articles out of the literature.

For research involving human participants, *PLOS ONE* has always required that submissions include an Ethics Statement reporting information about the study’s ethics approval and informed consent procedures. On occasion, journal staff have requested ethics approval documents for studies where there were concerns about adherence to the policy.

Several observations led to our decision to require ethics approval documents at the point of submission. Recently, the PLOS Publication Ethics team has handled higher volumes of cases where ethics documents received during their investigations raised concerns about whether ethics standards were upheld during the research process, whether measures were in place to protect participants in the research, or whether the reported findings were reliable. In light of these concerns, *PLOS ONE* ran a trial in 2022 wherein cohorts of authors were asked to supply ethics approval documents before peer review. Compliance was high, but what journal staff found was deeply troubling: in one cohort, nearly two-thirds of submissions did not meet *PLOS ONE*’s human subjects research requirements and were therefore rejected. Importantly, journal staff would not have detected the issues had they not requested the ethics documentation.

Given these observations, we updated the *PLOS ONE* Human Subjects Research Policy effective 1 March 2023 [1]. Under the updated policy, authors are required to provide original ethics approval documentation at the time of submission. These documents are evaluated by journal staff before peer review, but are not published. If there are any concerns about the ethics approval documents, if they are not provided, or if they indicate the study did not comply with our policies, the manuscript is rejected without external review. Per our longstanding policy, we will continue to request ethics documents for manuscripts submitted before March 2023 if deemed necessary by journal staff or editorial board members.

In the three months since we implemented the new policy, the proportion of manuscripts rejected by staff editors before review has increased by 30%. This statistic suggests that the updated policy removes studies performed without documented ethics approval from consideration at an early stage of the review process. With ever-increasing numbers of manuscripts published each year, implementing robust triage criteria at the start of the submission process helps ensure that Academic Editors and reviewers are asked to review only the work that meets core requirements outlined in journal policies.

As with any change, there have been challenges associated with the implementation of this new policy. We have found that many *PLOS ONE* authors are not (yet) familiar with our updated requirements, which has led to manuscripts being returned to authors at the point of initial submission so that they can provide the required documents on resubmission. We are looking for solutions to make this process more streamlined for authors, as well as to increase awareness about our updated policy. In addition, ethics approval documents vary by institution, and ethics standards can differ on a country-by-country basis. To ensure that these considerations are reflected in our processes and editorial decisions, we have provided additional training and support to the subject matter experts who examine the ethics documents during the initial editorial checks of new submissions [9].

Researchers with whom we have discussed the policy understand the rationale and are supportive of the updated requirements. Our submission volume has not been adversely affected. Moreover, at this early stage, we do not anticipate that the policy will have a significant effect on the number of papers that *PLOS ONE* publishes, either, despite the increased desk rejection rate.

We recognize the role that journals have in supporting the integrity of the published literature. We are proud to be taking this step to ensure that research submitted to *PLOS ONE* that involves human participants meets high ethics standards, so that the community can continue to trust, reuse, and build upon the work that we publish.
